# A Randomized Placebo Controlled Clinical Trial to Determine the Impact of Digestion Resistant Starch *MSPrebiotic*^®^ on Glucose, Insulin, and Insulin Resistance in Elderly and Mid-Age Adults

**DOI:** 10.3389/fmed.2017.00260

**Published:** 2018-01-22

**Authors:** Michelle J. Alfa, David Strang, Paramjit S. Tappia, Nancy Olson, Pat DeGagne, David Bray, Brenda-Lee Murray, Brett Hiebert

**Affiliations:** ^1^St. Boniface Research Centre, Winnipeg, MB, Canada; ^2^Department of Medical Microbiology, University of Manitoba, Winnipeg, MB, Canada; ^3^Deer Lodge Centre, Winnipeg, MB, Canada; ^4^Cardiac Sciences Program, I.H. Asper Clinical Research Institute, Winnipeg, MB, Canada

**Keywords:** digestion resistant starch, elderly, diabetes, glucose, insulin-resistance, gut microbiome, prebiotics

## Abstract

**Introduction:**

Type 2 diabetes (T2D) has reached epidemic proportions in North America. Recent evidence suggests that prebiotics can modulate the gut microbiome, which then plays an important role in regulating lipid metabolism, blood glucose, and insulin sensitivity. As such, prebiotics are appealing potential therapeutic strategies for prediabetes and T2D. The key objectives of this study were to determine the tolerability as well as the glucose and insulin modulating ability of *MSPrebiotic*^®^ digestion resistant starch (DRS) in healthy mid-age (MID) and elderly (ELD) adults.

**Materials and methods:**

This was a prospective, blinded, placebo-controlled study. Prediabetes and diabetes were among the exclusion factors. ELD (>70 years) and MID (30–50 years) Canadian adults were recruited and, after 2 weeks of consuming placebo, they were randomized to consume 30 g of either *MSPrebiotic*^®^ or placebo per day for 12 weeks. In total, 42 ELD and 42 MID participants completed the study. Blood samples were collected over the 14-week study and analyzed for glucose, lipid profile, and CRP, lipid particles, TNF-α, IL-10, insulin, and insulin resistance (IR).

**Results:**

At baseline, the ELD population had a significantly higher percentage (*p* < 0.01) with elevated glucose and significantly higher TNF-α (*p* < 0.01) compared to MID adults. *MSPrebiotic*^®^ DRS was well tolerated in both MID and ELD adults. There was a significant difference over time in blood glucose (*p* = 0.0301) and insulin levels (*p* = 0.009), as well as IR (HOMA-IR; *p* = 0.009) in ELD adults who consumed *MSPrebiotic*^®^ compared to placebo. No significant changes were found in MID adults.

**Conclusion:**

Our results suggest that dietary supplementation with prebiotics such as *MSPrebiotic*^®^ may be part of an effective strategy to reduce IR, a major risk factor for developing T2D, in the ELD.

**Clinical Trial Registration:**

NCT01977183 listed on NIH website: ClinicalTrials.gov, The metadata generated in this study have been submitted to the NCBI Sequence Read Archive (http://www.ncbi.nlm.nih.gov/bioproject/381931).

## Introduction

A prebiotic substance has been recently defined as “a substrate that is selectively utilized by host microorganisms conferring a health benefit.” (1). In view of the emerging link between particular gut microbiota ecosystems and the development of obesity and type 2 diabetes (T2D), prebiotics, along with probiotics, have received much attention in recent years (2–23).

Most of the published data on prebiotics relate to the effects of inulin, fructooligosaccharides (FOS), galactooligosaccharides (GOS), xylooligosaccharides (XOS), and lactulose (3, 4, 12, 24–26). These ingredients have been well tested *in vitro*, in animal studies, and occasionally in human studies with relatively consistent data. The characterization of these commercially available prebiotics is now reasonably well established. In contrast, recent publications (13, 27, 28) indicate that there are very few studies involving various categories of digestion resistant starch (DRS) in humans. The applicability of findings from animal studies, particularly those done in rodents, to human lipid metabolism has also been questioned (4, 13, 22). According to various authors (29–33), the pig is considered a better model than rodents, especially for studying age-related disorders in humans (31). Recent findings emanating from studies in swine suggest that DRS improves glycemic control and modifies blood lipoprotein profiles even at a relatively modest daily dose of 15 g (10.5 g of active ingredient) (33).

Reviews of recent evidence regarding the role of the gut microbiome in obesity, T2D, and metabolic disease in humans found several reports of gut dysbiosis (imbalance in the diversity or abundance of gut microbes) in patients with T2D compared to those without T2D (9, 13, 22). However, there were conflicting conclusions regarding the basis of the dysbiosis as data interpretation is confounded by different study methods, genetics, subject ethnic origins, diet, and geography (9). Furthermore, it is unclear whether the use of probiotics or prebiotics could modify this gut microbiome dysbiosis. There are substantial data to suggest that prebiotics can improve fasting blood glucose levels (6, 9, 13, 18, 34–36). However, human clinical studies often combine probiotic and prebiotic consumption making it difficult to assess the impact of prebiotics alone, and none of these published studies included elderly (ELD) adults (70 years or older).

Although many regulatory bodies around the world recognize the value of adequate dietary fiber, there are no dietary recommendations specifically for prebiotic consumption (7). Verbeke et al. (37) and Bindels et al. (12) have highlighted the value of fermentation metabolites as markers for prebiotic health benefits. However, this is difficult when there are no defined “healthy” levels of such metabolites, and current testing methods do not take into consideration the rapid absorption of such metabolites (e.g., short chain fatty acids). Although many of the health effects of probiotics and prebiotics have been linked to modulation of the gut microbiome, a limited number of clinical trials have been conducted in humans investigating the tolerability or function of DRS as a prebiotic (4, 11, 13, 15, 17, 28, 35, 38). Our recent publication focused on the microbiome changes in both mid-age (MID) and ELD adult groups after 3 months of consuming *MSPrebiotic*^®^ DRS. Our results demonstrated that no changes were observed in MID or ELD groups consuming the placebo (38). However, the MID and ELD groups consuming *MSPrebiotic*^®^ experienced significant increases in *Bifidobacteria*, and the ELD group consuming *MSPrebiotic*^®^ experienced a reduction in the abundance of *Proteobacteria*. Both of these microbiome changes are thought to provide health benefits. However, there are still many gaps in our knowledge regarding the health benefits of DRS (13). It is particularly unclear if DRS is well tolerated in MID or ELD humans, and whether this type of prebiotic alone can influence inflammation, lipid metabolism, blood glucose levels, or insulin resistance (IR).

The primary objectives of this aspect of the clinical study were to extend the microbiome findings of Alfa et al. (38) to determine if consumption of 30 g of *MSPrebiotic*^®^ DRS per day for 12 weeks was well tolerated compared to placebo and whether it could alter systemic health markers such as the lipid profile, inflammatory markers, glucose, insulin levels, or IR in MID or ELD groups.

## Materials and Methods

### Power Analysis and Sample Size

For the prospective study design and using the outcome measures described in the study protocol, the statistical power analysis indicated that a total sample of *n* = 20 in each of four groups (i.e., 20 ELD on placebo and 20 ELD on MSP as well as 20 MID on placebo and 20 MID on MSP) would have power = 0.80 to detect a Cohen’s *F* effect size = 0.33. This effect size was chosen based on the hypothesis that there would be moderate differences (0.50 SD) between the placebo and treatment groups for the ELD participants and that there would be small differences (0.20 SD) between the placebo and treatment groups for the MID participants. Effect sizes were estimated from published studies for other types of prebiotics using similar outcome measures (gastrointestinal tolerance, e.g., flatulence, bloating, and abdominal pain, in addition to serum glucose, lipid parameters, and inflammatory markers, such as C-reactive protein and TNF-α). A nominal α = 0.05 and two-tailed tests were used in the power calculations.

### Clinical Study

This was a prospective, randomized, blinded, placebo-controlled study. Research and ethics approval was obtained from the University of Manitoba Research Ethics Board prior to implementation. This study protocol was reviewed and approved by Health Canada (Submission #188517; “Notice of Authorization” dated June 5, 2013) and was also listed on the NIH ClinicalTrials.gov website (Identifier: NCT01977183). The clinical study was carried out in accordance with the ethical standards of the University of Manitoba and Health Canada. All modifications of the protocol were reported to Health Canada and the University of Manitoba Research Ethics Board for approval prior to implementation. The allocation sequence to placebo or study product was based on computer-generated random numbers. For the institutionalized ELD, the facility study nurse contacted residents (with permission from the facility physician) to determine if they would agree to the research study nurse contacting them about this clinical trial. For all other participants, a commercial recruitment agency provided a list of participants that they had permission from to contact for clinical trials. Participants who met enrollment criteria and gave informed consent in accordance with the Declaration of Helsinki were enrolled by the study coordinator or study nurse who also sequentially assigned participants to placebo or study product based on the list of computer-generated randomized numbers. Care providers, trial participants, laboratory testing personnel, and data analysts were blinded to which arm participants were assigned. All information collected (hard copy reports and electronic databases) for the purpose of the study was kept in a locked and secured area, and participant identifiers were treated in accordance with the Personal Health Information Act of Manitoba. All information sent for statistical analyses was “de-identified” and had only a study number but no participant identifiers. The University of Manitoba Office, Research Quality Management unit performed a voluntary audit of the study in a process that was independent from the investigators and the study sponsors.

**Figure 1:**
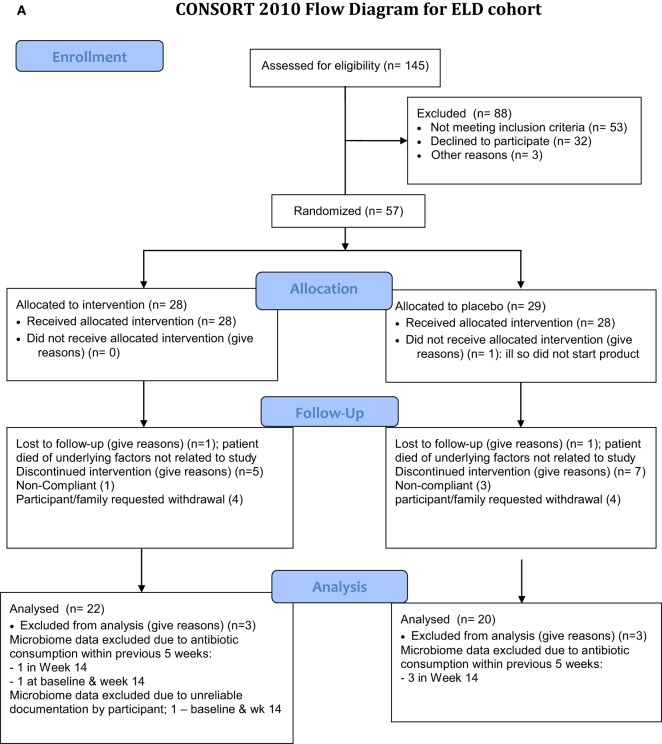

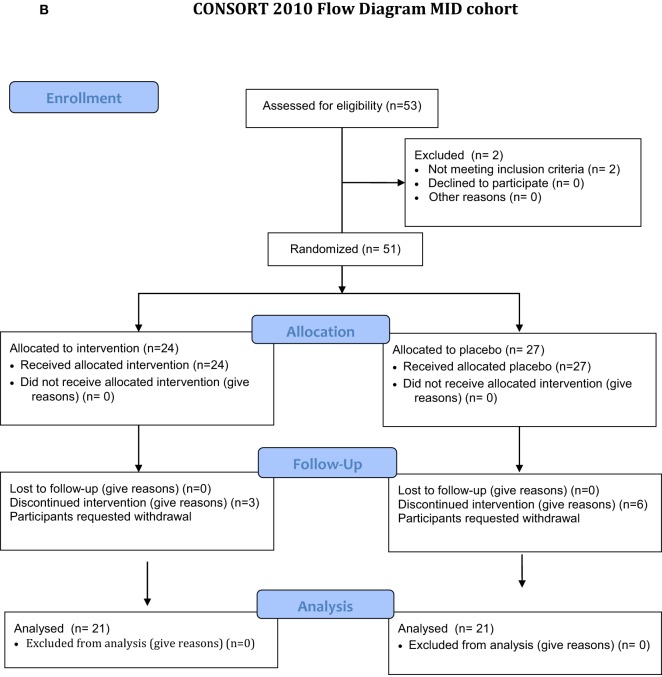
Flow chart of enrollment for the study. The elderly (ELD) enrollment is shown in **(A)** and the mid-age (MID) enrollment is shown in **(B)**. **(A)** Consort 2010 flow diagram for ELD cohort. **(B)** Consort 2010 flow diagram MID cohort.

All participants in this study were recruited in Winnipeg, MB, Canada. The ELD cohort was recruited from a long-term care (LTC) facility (11 completed the study), as well as from the community (31 completed the study), and consisted of adults >70 years old. The MID participants were recruited from the community and aged 30–50 years old (42 completed the study). The details of recruitment and dropout are shown in Figure 1. All participants (or authorized third party) provided written informed consent in compliance with the University of Manitoba informed consent guidelines. Participants were informed that they could request to withdraw from the clinical study at any time without any impact on their clinical care. To reduce confounding underlying factors unrelated to consumption of DRS, the exclusion criteria included the following: pregnancy, Crohn’s disease or any other inflammatory bowel disease, individuals with systemic lupus erythematosus, on cancer chemotherapy, prediabetes or diabetes, thyroid disease, renal disease, hepatic disease, previous gastrointestinal surgery (intestinal resection, gastric bypass, colorectal surgery), individuals on probiotics (e.g., probiotic yogurt), individuals on antibiotics at the time of recruitment or on antibiotics within the previous 5 weeks, individuals experiencing dysphagia, subjects using additional fiber supplements, and individuals on digestants, emetics, anti-emetics, medications for acid peptic disease, or taking antacids.

*MSPrebiotic*^®^ (MSPrebiotics Inc., Carberry, MB, Canada) DRS was used in this study. *MSPrebiotic*^®^ is to be consumed in unheated fluid or food products. This product is an unmodified natural DRS from potatoes, and the active ingredient is *Solanum tuberosum* extract, which is classified as a Natural Health Product on the Health Canada (39). It is composed of granules (15–100 µm in diameter) consisting of 80% amylopectin (branched glucose polymer with alpha-1,4 and alpha-1,6 linkages) and ~20% amylose (linear glucose polymer with mostly alpha-1,4 linkages). These DRS granules reach the colon relatively intact as they are not digested in the upper portions of the digestive tract. The DRS granules were analyzed using standardized validated methods by MSPrebiotic Inc. and demonstrated that *MSPrebiotic*^®^ contained 70% DRS.

Amioca TF (*Ingredion*™, Brampton, ON, Canada), a food-grade corn starch that is readily digestible, was the placebo in this study. The same standardized validated methods were used by MSPrebiotic Inc. to show that Amioca TF does not contain any DRS. All participants consumed 30 g of placebo daily for 2 weeks and then they were randomly assigned to either *MSPrebiotic*^®^ or placebo (30 g/day) for the remaining 12 weeks of the study. Participants were instructed to consume the study products at any time of the day providing it was taken 2 h before or after any other medication. In the LTC facility, *MSPrebiotic*^®^ or placebo was administered following the standard administration of medication protocol (2 h before or after receiving any other medication) using documented observed consumption as well as documentation in the daily health-log forms. The general population participants also documented product consumption by completing the daily health-log forms. In addition, the amount of returned product was documented by the study coordinator at monthly visits.

Both study products were provided to participants in identical sealed foil pouches labeled only with the information identifying that the product was part of a clinical trial. There was nothing regarding the packaging or labeling that would allow participants to know which product they were getting.

Participants completed a daily health-log to track excessive flatulence, changes in bowel movements, abdominal pain, and bloating. These parameters were used to assess tolerance of consuming *MSPrebiotic*^®^ versus placebo. The daily health log was also used to document consumption of the study product as well as the use of stool softeners and antibiotics. There were no changes to the normal daily diet consumed by participants other than the requirement that they did not consume probiotic-containing products.

Blood samples (fasting) were collected at enrollment (week 0), after 2 weeks of consuming placebo (participants were randomized to receive either *MSPrebiotic*^®^ or placebo at this time), and then at weeks 6, 10, and 14 (total of five samples collected). Blood samples were submitted to the laboratory for analysis on the day of collection. Additional blood samples collected at the same five time points were centrifuged, the serum was dispensed into aliquots, and stored at −70°C until sent for additional testing.

### Analysis Performed

#### Blood Analysis

Blood samples (fasting) at the five collection times indicated previously were analyzed by Diagnostic Services Manitoba, Winnipeg, MB, Canada, for C-reactive protein, glucose, and lipid profile using the standardized methods validated by the testing laboratory. In addition, aliquots of frozen serum (fasting serum) were sent to LipoScience Inc. (Raleigh, NC, USA) for lipid particle size analysis using NMR (40, 41), as well as tests to determine lipid profile, glucose level, insulin level, and IR. Samples were collected, transported, and analyzed as per the company instructions and protocols (www.labcorp.com). The frozen serum aliquots were also tested in-house for human TNF-α and IL-10 using commercial assay kits (Invitrogen, Frederick, MD, USA) as per the manufacturer’s instructions. Each frozen aliquot was thawed once for analysis and any unused portion of the aliquot was discarded.

#### Statistical Analysis

Baseline characteristics were compared between the MID and ELD cohorts using a Chi square test or Fisher’s exact test for categorical variables, and a Student’s *t*-test or Mann–Whitney test where appropriate. The difference in the change (baseline to 14 weeks) experienced in the control and *MSPrebiotic*^®^ groups were compared for several systematic markers, lipid measures, glucose, and IR *via* a non-parametric Mann–Whitney test. In addition, a repeated measures analysis of variance was also performed as a supplementary analysis on variables that were measured at more than two time points to obtain a group, time, and group/time effect. Data collected in the weekly health log were assessed *via* mixed-effects models containing random slopes and intercepts. All *p*-values calculated were two-tailed, and a *p*-value less than 0.05 was considered statistically significant. All statistical analyses were performed using SAS version 9.3.

## Results

Enrollment for the clinical study was started in September 2013 and completed in May 2015. Figure 1 outlines the recruitment, enrollment, and attrition of participants in the ELD and MID groups. In the ELD group, there were 31 participants from the community and 11 participants who were residents of LTC that completed the clinical trial. The data for non-institutionalized ELD participants and institutionalized ELD participants showed similar trends for all the parameters we evaluated, so the data from both ELD groups were combined for all subsequent analyses. All the MID participants enrolled were from the community (42 completed the clinical trial). The baseline (at the time of enrollment) levels of blood glucose, cholesterol parameters, and inflammatory markers of the ELD and MID groups are shown in Table 1. TNF-α levels were significantly higher (*p* < 0.01) in ELD versus MID at the time of enrollment, while C-reactive protein was elevated in ELD participants, but not significantly *(p* = 0.052). Furthermore, the percentage of participants with elevated blood glucose levels was significantly higher *(p* = 0.03) in the ELD group (10/42) compared to the MID group (2/42) at baseline despite all participants meeting enrollment criteria that excluded diabetics and prediabetics.

**Table 1:** Baseline Parameters for mid-age (MID) and elderly (ELD) groups.

Variable	Baseline MID*	Baseline ELD*	*p*-Value*
	Median (quartile 1–quartile 3)	Median (quartile 1–quartile 3)	
Number of participants	42	42	
Number females/males	24/18	25/17	0.82
Age	42 (37–47) [range: 32–50]	75 (73–82) [range: 70–96]	**<0.01**
Weight (kg)	78.4 (70.5–88.1)	76.7 (64.4–85.7)	0.17
Glucose^a^ (normal range; 3–6 mmol/L)	5.3 (4.9–5.6)	5.6 (5.0–5.9)	0.06
Cholesterol (desirable < 5.2 mmol/L)	5.0 (4.6–5.5)	5.1 (4.5–6.5)	0.40
Triglycerides (desirable < 1.7 mmol/L)	1.1 (0.9–1.7)	1.3 (0.9–1.8)	0.48
HDL cholesterol (desirable > 1.1 mmol/L)	1.5 (1.2–1.8)	1.4 (1.2–1.7)	0.83
LDL cholesterol (desirable < 3.4 mmol/L)	2.9 (2.5–3.4)	2.9 (2.4–3.9)	0.69
Total Chol/HDL (desirable < 4.5 mmol/L)	3.6 (2.7–4.4)	3.5 (2.9–4.5)	0.99
LDL/HDL (desirable < 3.5)	2.1 (1.5–2.7)	2.2 (1.5–2.5)	0.98
C-Reactive protein (desirable < 8 mg/L)	3.0 (1.9–4.8)	4.0 (2.7–6.6)	**0.04**
TNF-α (desirable < 2.1 pg/mL)	0.7 (0.0–1.3)	1.8 (1.0–2.5)	**<0.01**
IL-10 (desirable < 1 pg/mL)	0.0 (0.0–0.0)	0.0 (0.0–0.0)	0.86

*There was no significant differences in the data from institutionalized versus non-institutionalized ELD participants at baseline so the data have been pooled. Placebo was consumed by all participants for 2 weeks and then participants were randomized to continue on placebo or to consume 30 g/day *MSPrebiotic*^®^ for the next 12 weeks. All continuous values are expressed as median (quartile 1–quartile 3), compared using Mann–Whitney test. All categorical values are expressed as *N*/total, compared using Chi-square test or Fisher’s exact test.

*^a^Although there was no statistically significant difference in the median glucose level, there was a significant difference (*p* = 0.03) in the number of ELD at baseline who had elevated (i.e., above 6 mmol/L) glucose (10/42) compared to MID at baseline (2/42)*.

*Bold values represent p < 0.05*.

**Figure 2:**
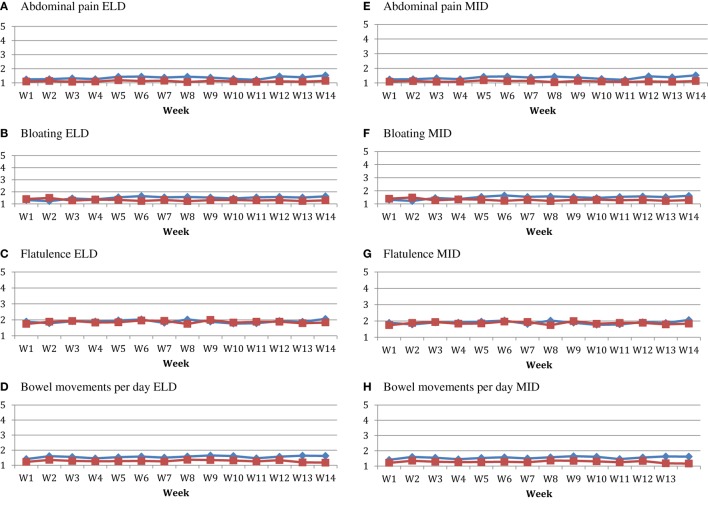
Tolerability of consuming *MSPrebiotic*^®^ versus Placebo over time in elderly (ELD) and mid-age (MID) adults. All ELD **(A–D)** and MID **(E–H)** participants consumed 30 g placebo/day for 2 weeks and then were randomized to continue placebo or consume 30 g *MSPrebiotic*^®^/day for the remaining 12 weeks of the study. For abdominal pain, bloating and flatulence the scale was from 1 (none) to 5 (extreme). The red square symbols represent the placebo group, and the blue diamond symbols represent the *MSPrebiotic*^®^ group. There was no statistically significant difference between groups for any of these parameters. **(A)** Abdominal pain ELD. **(B)** Bloating ELD. **(C)** Flatulence ELD. **(D)** Bowel movements per day ELD. **(E)** Abdominal pain MID. **(F)** Bloating MID. **(G)** Flatulence MID. **(H)** Bowel movements per day MID.

The compliance with consuming the study product was: MID-placebo; 93.5% (range: 25.0–100.0%; median: 93.5%), MID-*MSPrebiotic*^®^; 91.7% (range: 0.0–100.0%; median: 99.0%), ELD-placebo; 95.4% (range: 66.3–100.0%; median: 99.0%) and ELD-*MSPrebiotic*^®^; 97.2% (range: 80.6–100.0%; median: 99.0%). *MSPrebiotic*^®^ was well tolerated in both groups, as shown in Figure 2. Levels of abdominal pain, bloating, and flatulence were all low (2 or less on a scale of 1 = none to 5 = excessive) for both groups taking either placebo or *MSPrebiotic*^®^, and there were no significant differences over the study period. The mean number of bowel movements for the MID and ELD group on placebo versus *MSPrebiotic*^®^ did not change over the duration of the study, and at all times the mean number of bowel movements was less than two per day for both groups.

Supplementation with *MSPrebiotic*^®^ or placebo had no effect on systemic cholesterol transport or IL-10 for ELD or MID groups (Table 2). Lipid profile and particle size profile were also largely unaffected after consumption of *MSPrebiotic*^®^ versus placebo for ELD and MID (Tables 3 and 4, respectively). Only in the MID group consuming *MSPrebiotic*^®^ was there a significant increase in large VLDL and chylomicron particles (Table 4).

**Table 2:** Impact of consuming *MSPrebiotic*^®^ versus placebo^a^ on systemic markers in mid-age (MID) and elderly (ELD) adults.

	MID (14 weeks)			ELD (14 weeks)		
	Median (quartile 1–quartile 3)			Median (quartile 1–quartile 3)		
Variable	Control group (*N* = 21)	*MSPrebiotic*^®^ group (*N* = 21)	*p*-Value*	Control group (*N* = 20)	*MSPrebiotic*^®^ group (*N* = 22)	*p*-Value*
Glucose (normal range; 3–6 mmol/L)	5.1 (4.7–5.5)	5.1 (4.9–5.6)	0.5538	5.5 (5.0–5.9)	5.1 (4.8–5.5)	**0.0301**
Cholesterol (desirable < 5.2 mmol/L)	5.0 (4.4–5.1)	5.1 (4.7–5.8)	0.3382	5.2 (4.3–6.2)	5.3 (3.9–6.4)	0.4418
Triglycerides (desirable < 1.7 mmol/L)	1.2 (0.8–1.5)	1.4 (1.0–2.5)	0.2266	1.3 (1.1–2.1)	1.3 (0.9–1.8)	1.0000
HDL cholesterol (desirable > 1.1 mmol/L)	1.4 (1.2–1.8)	1.1 (1.0–1.4)	0.6298	1.5 (1.3–1.9)	1.4 (1.2–1.8)	0.9899
LDL cholesterol (desirable < 3.4 mmol/L)	2.7 (2.6–3.1)	3.2 (2.4–3.5)	0.5129	2.8 (2.3–4.3)	3.2 (2.2–3.9)	0.6960
Total Chol/HDL (desirable < 4.5 mmol/L)	3.3 (2.6–4.4)	4.4 (3.2–5.2)	0.4488	4.0 (2.4–5.2)	3.4 (2.8–4.3)	0.9397
LDL/HDL (desirable < 3.5)	2.0 (1.4–3.0)	2.6 (1.7–3.1)	0.5563	2.5 (1.2–3.2)	2.0 (1.5–2.6)	0.9094
CRP (desirable ≤ 8 mg/L)	3.1 (1.9–4.9)	3.9 (2.0–7.3)	0.2609	2.6 (1.8–4.2)	4.1 (2.7–6.1)	0.9792
TNF-α (desirable ≤ 2.1 pg/mL)	0.9 (0.0–1.5)	0.0 (0.0–1.7)	0.6897	1.6 (1.0–2.7)	1.7 (0.9–2.3)	0.6503
IL-10 (desirable ≤ 1 pg/mL)	0.0 (0.0–0.0)	0.0 (0.0–0.0)	1.0000	0.0 (0.0–0.0)	0.0 (0.0–0.0)	1.0000

*^a^Placebo was consumed by all participants for 2 weeks, and then participants were randomized to continue on placebo or to consume 30 g/day *MSPrebiotic*^®^ for the next 12 weeks. All continuous values are expressed as median (quartile 1–quartile 3). Summary statistics reflect measurements obtained at 14-week time point*.

*p-Values reflect the difference in the change (baseline–14 weeks) experienced in the control and *MSPrebiotic*^®^ groups. *p*-Values were calculated using a Mann–Whitney test.

**Table 3:** Lipid particle and insulin resistance (IR) profiles in elderly after consumption of *MSPrebiotic or* placebo^a^.

	Baseline		14 weeks		*p*-Value*, control versus *MSPrebiotic*^®^
	Median (quartile 1–quartile 3)		Median (quartile 1–quartile 3)		
	Control group	*MSPrebiotic*^®^ group	Control group	*MSPrebiotic*^®^ group	
VLDL and chylomicron particles (total)	49.4 (19–85.5)	43.8 (34.4–84)	36 (22.9–78.8)	44 (20.7–91.9)	0.9198
Large VLDL and chylomicron particles	2.3 (2–3.7)	2.5 (1.3–3.5)	3 (1.2–6.6)	1.7 (0.8–3.4)	0.4964
Medium VLDL particles	21.8 (7.5–47.1)	20.3 (12.2–33.1)	17.9 (11.4–46.6)	19.3 (13.2–52.6)	0.5289
Small VLDL particles	17.2 (7.7–28.6)	21.6 (11.2–36.2)	16.9 (4.9–31.2)	20.7 (4.2–38.2)	0.9198
LDL particles (total)	1200.5 (1,050–1,493)	964 (884–1,299)	1,233 (978.5–1592.5)	1094.5 (953–1,374)	0.8012
IDL particles	68.5 (44.5–94.5)	47 (29–114)	83 (58.5–131.5)	74 (41–119)	0.8305
Large LDL particles	596 (348.5–878.5)	581 (416–775)	466 (257–730)	516.5 (406–849)	0.2267
Small LDL particles (total)	581.5 (113.5–804.5)	393.5 (201–508)	666 (312.5–988)	419.5 (264–691)	0.6323
HDL particles (total)	34.1 (26.4–39.9)	31 (27.8–37.3)	33.2 (27.6–37.9)	31.6 (27–36)	0.3137
Large HDL particles	6.2 (3.2–8.4)	5.5 (4.5–8.3)	5.1 (3.1–9.4)	5.5 (3.6–8.5)	0.4276
Medium HDL particles	7.1 (3.5–11.2)	6.6 (4.4–10.3)	8.1 (3.8–12.5)	7.9 (5.1–11.1)	0.8511
Small HDL particles	19.5 (14–21.4)	18.7 (14.8–21.7)	19.7 (14.3–23.2)	17.5 (14.4–20.1)	0.1043
VLDL size	47.8 (44.1–52.4)	46.3 (42.9–50.7)	47.7 (43.4–54.3)	47.4 (42.9–51.4)	0.6473
LDL size	21.6 (20.7–22.3)	21.8 (21.5–22.3)	21.3 (20.4–21.9)	21.8 (21–22)	0.6564
HDL size	9.5 (8.9–10.1)	9.5 (9.2–9.8)	9.4 (8.7–9.7)	9.5 (9.1–9.7)	0.2441
Triglyceride (total)	103 (80–162)	99 (82–145)	92 (85–174)	97.5 (76–165)	0.4129
VLDL and chylomicron triglyceride (total)	70.9 (36.5–115.7)	61.1 (49–94)	54.5 (41.9–141.6)	59.6 (40.7–134.9)	0.7150
HDL cholesterol (total)	47.5 (44–67.5)	49.5 (40–57)	49 (42.5–65)	46 (40–60)	0.5869
Insulin μIU/mL (range: 2.6–24.9)	8.1 (4.8–12.7)	9 (5.5–15.5)	7.4 (5.8–13)	8.1 (5.8–10.7)	**0.0091**
Glucose mmol/L (normal range: 3–6 mmol/L)	5.8 (5.4–6.2)	5.8 (5.3–6.1)	5.8 (5.3–6.2)	5.5 (5.2–5.8)	**0.0321**
LP-IR (range: 0–100)	37 (32–54)	38.5 (26–48)	42 (30.5–65)	31 (23–44)	0.2839
HOMA-IR	38.5 (20.5–61)	40.5 (23.6–67.5)	32.3 (25.2–63.2)	35.1 (25.7–46.1)	**0.0095**
QUICKI-IR	0.34 (0.32–0.38)	0.34 (0.31–0.37)	0.35 (0.32–0.36)	0.35 (0.33–0.36)	**0.0035**

*^a^Placebo was consumed by all participants for 2 weeks and then participants were randomized to continue on placebo or to consume 30 g/day *MSPrebiotic*^®^ for the next 12 weeks. All continuous values are expressed as median (quartile 1–quartile 3)*.

*p-Values reflect the difference in the change (baseline–14 weeks) experienced in the control and *MSPrebiotic*^®^ groups. *p*-Values were calculated using a Mann–Whitney test.

*Bold values represent p values < 0.05*.

**Table 4:** Lipid particle and insulin resistance (IR) profiles in mid-age after consumption of *MSPrebiotic or* placebo^a^.

	Baseline		14 weeks		*p*-Value*, control versus *MSPrebiotic*^®^
	Median (quartile 1–quartile 3)		Median (quartile 1–quartile 3)		
	Control group	*MSPrebiotic*^®^ group	Control group	*MSPrebiotic*^®^ group	
VLDL and chylomicron particles (total)	43.6 (23.2–61.6)	38.7 (23.4–69.5)	46.6 (21.1–68.1)	54.5 (28.4–98.6)	0.3585
Large VLDL and chylomicron particles	2.7 (1.5–4.7)	3.1 (2–5.9)	3 (2–5.4)	4 (3–6.3)	**0.0200**
Medium VLDL particles	16.6 (9.7–34.7)	23.1 (10–40.3)	17.8 (9.7–33.1)	19.6 (15.5–49.7)	0.5050
Small VLDL particles	18.7 (11.1–27.6)	13.2 (9.6–27.2)	18.2 (10.2–28.8)	19.2 (9.4–30.8)	0.8111
LDL particles (total)	1,059 (823–1,274)	1,137 (1,069–1,371)	986 (832–1,178)	1,189 (926–1,422)	0.5886
IDL particles	101 (74–137)	80 (46–148)	86 (65–143)	88 (60–117)	0.9099
Large LDL particles	484 (332–521)	433 (363–564)	423 (379–491)	488 (335–604)	0.7153
Small LDL particles (total)	505 (156–699)	584 (399–907)	439 (171–735)	595 (438–875)	0.4281
HDL particles (total)	34.3 (31–36.2)	36.4 (30.9–38.5)	34 (30.9–39)	34.3 (28.6–37.2)	0.1073
Large HDL particles	5.3 (2.7–9.3)	4.5 (2.5–7.4)	4.7 (2.9–8.2)	3.8 (2.3–6.1)	0.5628
Medium HDL particles	9.1 (6.3–11.3)	13.8 (9.3–16.8)	9.2 (6.7–13.8)	14.4 (9–18.5)	0.1552
Small HDL particles	18.5 (15.6–20.5)	16.4 (12.8–17.7)	18.2 (15.3–20.9)	14.3 (12.8–18.2)	0.9298
VLDL size	50.6 (45.1–53.1)	51.3 (45.9–56.7)	50.2 (45.7–54)	50.3 (46.8–54.8)	0.9676
LDL size	21.4 (21.1–22.1)	21 (20.7–21.8)	21.7 (20.8–22.3)	21.2 (20.5–21.8)	0.6502
HDL size	9.3 (8.8–9.8)	9.1 (8.7–9.6)	9.3 (8.8–9.6)	9.1 (8.7–9.6)	0.4634
Triglyceride (total)	92 (76–125)	97 (81–158)	97 (75–135)	111 (88–225)	0.1217
VLDL and chylomicron triglyceride (total)	63.7 (38.3–85)	59.8 (38.9–124.8)	63.1 (46–101.4)	78 (51.5–181.4)	0.0761
HDL cholesterol (total)	50 (47–61)	49 (41–62)	47 (41–62)	47 (41–56)	0.5039
Insulin μIU/mL (range; 2.6–24.9)	12.9 (9.2–17)	14.1 (9.3–31)	11.8 (8.5–15)	16.5 (9.2–27.1)	0.3431
Glucose mmol/L (normal range; 3–6 mmol/L)	5.2 (4.8–5.6)	5.4 (5.1–5.6)	5.0 (4.8–5.3)	5.2 (5.0–5.6)	0.5702
LP-IR (range: 0–100)	42 (27–58)	60 (35–65)	45 (27–61)	57 (29–69)	0.9098
HOMA-IR	55.3 (38–70.3)	56.1 (42.6–141.9)	47.8 (34.5–75.4)	69.4 (38.4–112.4)	0.2727
QUICKI-IR	0.32 (0.31–0.34)	0.32 (0.29–0.34)	0.33 (0.31–0.35)	0.31 (0.29–0.34)	0.4088

*^a^Placebo was consumed by all participants for 2 weeks and then participants were randomized to continue on placebo or to consume 30 g/day *MSPrebiotic*^®^ for the next 12 weeks. All continuous values are expressed as median (quartile 1–quartile 3)*.

*p-Values reflect the difference in the change (baseline–14 weeks) experienced in the control and *MSPrebiotic*^®^ groups. *p*-Values were calculated using a Mann–Whitney test.

*Bold values represent p values < 0.05*.

**Figure 3:**
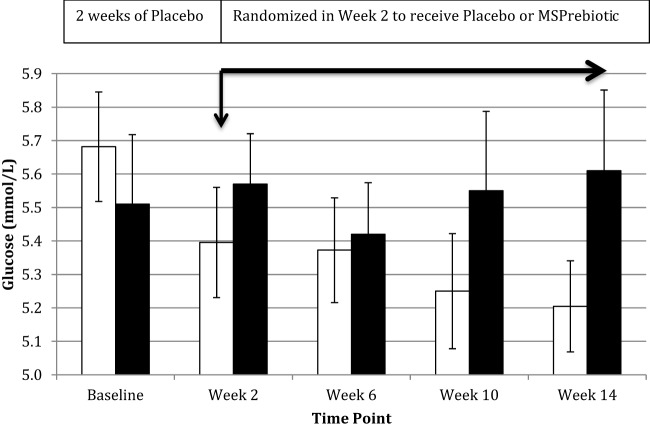
Impact of *MSPrebiotic*^®^ versus placebo on mean blood glucose levels in the elderly (ELD) group. Placebo (30 g/day) was consumed by all participants for 2 weeks and then participants were randomized to continue on placebo (30 g/day) or to consume 30 g/day *MSPrebiotic*^®^ for the next 12 weeks. Serum samples over the course of the study for the ELD group were analyzed on the day of collection for mean glucose levels. The black bars represent the placebo group and the white bars represent the *MSPrebiotic*^®^ group. There was a significant group/time interaction for placebo versus *MSPrebiotic*^®^ for the samples taken in week 0 compared to week 10 (8 weeks of consuming *MSPrebiotic*^®^) or week 14 (12 weeks of consuming *MSPrebiotic*^®^), *p* = 0.045 and *p* = 0.0124, respectively.

Despite all participants being screened against exclusion criteria for diabetes or prediabetes, the ELD group had significantly more individuals with elevated blood glucose levels at baseline (Table 1). Blood glucose levels of ELD participants assigned to take *MSPrebiotic^®^* were not significantly different from ELD participants assigned to take placebo at baseline (*p* = 0.515). Blood glucose levels significantly differed over time (*p* = 0.0301) in ELD participants taking *MSPrebiotic*^®^ for 12 weeks compared to those consuming the placebo (Table 2). The change over the 14-week study is shown in Figure 3. Retesting of the samples at a second independent laboratory confirmed these results (Tables 3 and 4). Furthermore, the group/time interaction in the ELD population taking *MSPrebiotic*^®^ versus placebo (Figure 3) showed there was a significant reduction (*p* = 0.045) in glucose levels as early as week 10 (i.e., after 2 weeks of placebo and 8 weeks of *MSPrebiotic*^®^).

Blood insulin levels also significantly differed over time in ELD participants consuming *MSPrebiotic*^®^ compared to the placebo group (Table 3). IR was calculated using three methods: LP-IR (calculated by LipoScience), HOMA-IR (40, 41), and QUICKI-IR (40) (Tables 3 and 4). The LP-IR IR was not significantly different between *MSPrebiotic*^®^ and placebo groups over time in either ELD or MID categories (Tables 3 and 4). However, HOMA-IR and QUICKI-IR values showed significant improvement over time in IR for participants on *MSPrebiotic*^®^ compared to placebo (*p* = 0.009 and 0.004, respectively).

## Discussion

There is a strong link between gut microbiota dysbiosis, where pro-inflammatory *Proteobacteria* abundance increases, and the endotoxin from these bacteria stimulates TLR4 to cause an increase in inflammatory markers (e.g., TNF-α) and an increase in IR, which over a prolonged period can lead to hyperglycemia and ultimately T2D (5). We previously demonstrated that *MSPrebiotic*^®^ promotes the growth of *Bifidobacteria* and corrects the *Proteobacteria* dysbiosis in the ELD (38), meeting recent prebiotic classification criteria (1). Here, we extend our analysis of our 2017 clinical trial by demonstrating that *MSPrebiotic*^®^ is well-tolerated in MID and ELD adults (Figure 2), and by further characterizing the physiological consequences of the “bifidogenic effect.” Importantly, we found that *MSPrebiotic*^®^ significantly reduced blood glucose and insulin levels by 7 and 41%, respectively, in otherwise healthy ELD individuals (Table 3). Taken together, the results from our clinical study suggest that *MSPrebiotic*^®^ may be an effective tool in promoting the growth of *Bifidobacteria* and improving blood glucose management, especially in the ELD.

Previous studies have examined the blood glucose-lowering and insulin-sensitizing potential of DRS prebiotics (11, 13, 15, 21, 34). These studies examined the role of DRS as a carbohydrate replacement in food rather than as a supplement, where at least some of the effects on blood glucose level can be attributed to differences in the amount of digestible starch. While connections between DRS consumption and improved insulin sensitivity have been reported, they do not necessarily translate to lower blood glucose levels (15, 18, 19). In healthy people, this should not be surprising, given that the body will adapt by generating glucose *via* gluconeogenesis to prevent hypoglycemia in those with proper glycemic control (11). Such is likely the case in the MID population, where we observed no effect of *MSPrebiotic*^®^ on blood glucose, insulin levels, or IR (Table 4). However, in the ELD population, where the baseline level of glucose was elevated toward the high end of the normal range (Table 1), consumption of *MSPrebiotic*^®^ lowered the blood glucose levels within 8 weeks of supplementation (Figure 3). Gower et al. (18) reported that there was no impact of maize-derived DRS2 consumption on IR in women with adequate glucose homeostasis, whereas there was a significant reduction in IR in women with elevated IR. Our data are therefore consistent with those of Gower et al. (18), extending these findings to both men and women who are MID or ELD. In combination with other published data (2, 10, 13, 22, 33), our data suggest that various types of DRS prebiotics, just like other types of oligosaccharide prebiotics (i.e., FOS, GOS, XOS, etc.), have differing abilities to modulate lipids, glucose, and IR. Furthermore, the ability of prebiotics to modulate these parameters appears to be dependent on the extent of elevated blood glucose and IR (13, 18). For example, DRS can improve IR in humans who are prediabetic (18), but not after T2D has been established (13). Although the mean reduction of glucose in the ELD on *MSPrebiotic*^®^ was small (7%), this is likely due to the limited room for blood glucose improvement in this group. For this reason, our findings support the value of future clinical studies on *MSPrebiotic^®^* in prediabetics.

Our data showed that *MSPrebiotic*^®^ significantly reduced blood glucose and insulin levels in the ELD population and reduced IR as measured by HOMA-IR and QUICKI-IR (Table 3). However, LP-IR, which includes lipid particle analysis to determine IR in prediabetics with abnormal lipid profiles (40), was not significantly affected, suggesting that lower blood glucose levels were due to improved use of endogenous insulin rather than improved lipid metabolism. Given that the ELD and MID populations had neither prediabetes nor abnormal lipid profiles, it is not surprising that the HOMA-IR and QUICKI-IR (measures of IR based solely on fasting blood glucose and insulin) showed significant improvements in IR for those consuming *MSPrebiotic*^®^ compared to placebo but LP-IR was not different. The microbiome changes and increases in butyrate (38), combined with lower blood glucose, insulin, and IR, were all benefits achieved without the use of probiotic supplementation. As suggested by others (5, 9, 13, 20, 22, 26, 28), our data support the concept that it is possible for the gut microbiome to be modulated *via* the consumption of specific types of prebiotics, leading to better glycemic control especially in ELD adults. Further studies are needed to clarify if the glucose and IR changes that were achieved by *MSPrebiotic*^®^ consumption by the ELD and MID as described in this study are linked to or are independent from the bifidogenic effect also achieved by *MSPrebiotic*^®^ in this same group of participants (38).

A recent animal study evaluated the effect of *MSPrebiotic*^®^ supplementation on blood glucose, insulin, and IR in a swine model fed a Western diet (33). Of note, the swine were fed one half of the daily amount (15 g) of *MSPrebiotic*^®^ that was consumed by our participants (30 g). While insulin levels were not significantly affected, blood glucose levels and IR were both reduced in swine consuming *MSPrebiotic*^®^. Furthermore, the authors also found a 141% increase in glucagon-like peptide-1 (GLP-1) in swine supplemented with *MSPrebiotic*^®^. GLP-1 is an incretin secreted by enteroendocrine cells (L-cells) in the colon in response to bacteria metabolites, which improves insulin utilization (8, 13), and increased GLP-1 could explain improved insulin sensitivity in response to fermentation of *MSPrebiotic*^®^ in swine.

While determining the mechanisms by which blood glucose and IR decreased in the ELD group consuming *MSPrebiotic*^®^ is beyond the scope of our investigation, it is appealing to speculate that increased GLP-1 in the *MSPrebiotic*^®^ ELD group contributes to the improved glycemic response, which is consistent with recent publications (13, 23, 33). Alternatively, fermentation of *MSPrebiotic*^®^ may have improved IR by stimulating production of short-chain fatty acids such as butyrate that have been shown to improve glucose homeostasis through intestinal gluconeogenesis (16). Such an explanation would also be consistent with our previous microbiome publication demonstrating that consumption of *MSPrebiotic*^®^ led to a significant increase in the relative abundance of butyrate in ELD individuals (38). Other currently unknown mechanisms may also be at work (5, 8). Future studies examining the mechanisms by which *MSPrebiotic*^®^ improves glycemic response in animals and humans are warranted.

Rideout et al. (33) also examined markers of cholesterol metabolism in their Western diet swine model, where they found that supplementation with *MSPrebiotic*^®^ led to significant increases in total HDL particles, driven largely by an increase in the small HDL subclass of particles. Other cholesterol parameters were unchanged. The effect of DRS on cholesterol has been examined elsewhere, for example, in prediabetic obese individuals, where DRS improved these parameters (17). The consumption of wheat-based chemically modified resistant starch was correlated with a significant reduction in total cholesterol, HDL, and non-HDL (including LDL), but had no significant effect on triglycerides (2). Other published data (2, 10, 13) suggest that different types of DRS have differing abilities to modulate lipids, glucose, and IR. *MSPrebiotic*^®^ had no significant impact on total cholesterol, HDL, or LDL, in either MID or ELD participants. However, there was a significant increase in the number of large VLDL/chylomicron particles in the MID group. To our knowledge, this is the first time that the consumption of DRS has been associated with such an effect, and the clinical significance of this is unclear.

Our data extend the published literature with respect to the impact of prebiotic consumption on the understudied ELD demographic (42, 43), which represents a large proportion of the Canadian population. Others have reported that ELD adults have an increased prevalence of non-specific inflammatory markers compared to younger adults, and this is thought to be due to increased permeability of the gut mucosa (5, 11, 14, 36, 42). Our data support this concept, as there were more ELD participants with elevated baseline CRP (trend toward statistical significance; *p* = 0.08) and TNF-α (*p* < 0.01) compared to the MID participants. This is likely linked to the microbiome dysbiosis in our ELD group, who at baseline had significantly increased levels of pro-inflammatory *Proteobacteria* (*Escherichia coli/Shigella*) (38). Of interest, the elevated inflammatory levels in the ELD group were not reduced by the end of the study in either the placebo or *MSPrebiotic*^®^ groups. This suggests that the colonocyte apoptosis or reduced mucus production (36, 44) leading to increased bowel permeability (thought to be the basis of increased inflammatory response) could be irreversible or take longer than the 3-month period evaluated in our study. It may be that earlier addition of prebiotics to the diet (i.e., before 70 years old) is needed to prevent the bowel damage, permeability, and the associated increase in non-specific inflammatory markers.

A limitation of our study was the relatively small sample size (*N* = 21 or 22 for each group). However, we offset this limitation by collecting samples at multiple time points over 14 weeks for each participant (i.e., not just a one-time sampling). Despite a small sample size, we were able to demonstrate significant changes in glucose, insulin, and IR, supporting the adequacy of our power calculations. Furthermore, we would recommend future studies that have less extensive exclusion criteria than the current study undertaken in the ELD population to determine the impact of DRS in a wider cross-section of this demographic. This type of clinical study is needed to more extensively assess the clinical impact of *MSPrebiotic*^®^ in the ELD population.

In conclusion, our data demonstrated that *MSPrebiotic*^®^ is a prebiotic as defined by Gibson et al. (1) that is well-tolerated in Canadian adults and that this DRS effectively lowers blood glucose and insulin levels and reduces IR in ELD adults. Future studies in subjects with prediabetes and T2D are warranted given that dietary supplementation is an understudied avenue for the amelioration of risk factors linked to the development and progression of these conditions. While *MSPrebiotic*^®^ had a minimal impact on cholesterol metabolism, further studies in dyslipidemic individuals are required to fully evaluate the effect(s) of this DRS on these measures. Consumption of *MSPrebiotic*^®^ for 3 months was not sufficient to reduce the elevated CRP and TNF-α levels in the ELD group, suggesting that earlier intervention (i.e., before age 70) may be necessary to prevent the development of gut barrier damage or that these inflammatory markers are due to other underlying factors such as vascular comorbidities (45).

## Ethics Statement

This study was carried out in accordance with the recommendations of University of Manitoba Research Ethics Board and Health Canada with written informed consent from all subjects. All subjects gave written informed consent in accordance with the Declaration of Helsinki. The protocol was approved by the University of Manitoba Research Ethics Board and Health Canada. No animal subjects were used in this study.

## Author Contributions

All the authors provided substantial contribution to the design of the work as well as the acquisition, analysis, and interpretation of the work. MA drafted the manuscript, and all the other authors contributed to revising and critical assessment of intellectual content. All the authors have given final approval of this manuscript and agree to be accountable for all aspects of the work and to ensuring that all questions related to the accuracy or integrity of any part of the work are properly investigated and resolved.

## Conflict of Interest Statement

MSPrebiotics Inc. paid for printing of a poster and for flight, accommodation, and speaker honorarium for MA to attend 4th Microbiome R&D and Business Collaboration Forum: USA October 3–4, 2016, San Diego, CA, USA, for poster presentation of the microbiome data from this clinical study (i.e., data presented in this current manuscript were not presented at the conference). There are no disclaimers or conflicts of interest for any of the other authors.
